# Gene silencing of heparanase results in suppression of invasion and migration of hepatoma cells

**DOI:** 10.1186/1477-7819-12-85

**Published:** 2014-04-04

**Authors:** Weiwei Dong, Huixia Zhao, Caihong Zhang, Paili Geng, Sarengaowa, Qiuwen Li, Jianhua Zhu, Guanghui Li, Shufang Zhang, Ming Ye, Wenhua Xiao

**Affiliations:** 1 Department of Oncology, First Affiliated Hospital, Chinese PLA General Hospital, Beijing 100048, China; 2 Department of oncology, Chinese PLA general hospital, Beijing, China; 3 Department of Immunology, Medical College of Qinghai University, No 16 Kunlun Road, Xining, Qinghai Provence 810001, China; 4 Department of Histology and Embryology, Inner Mongolia Medial College, Hohhot, Inner Mongolia Autonomous Region 010010, China

**Keywords:** transcriptional gene silencing, heparanase gene, hepatoma cell, invasion, migration

## Abstract

**Background:**

This study investigated the effect of transcriptional gene silencing (TGS) of the heparanase gene on hepatoma SMCC-7721 cells.

**Methods:**

SiRNAs targeting the promoter region and coding region of the heparanase gene were designed and synthesized. Then the siRNAs were transfected into hepatoma SMCC-7721 cells by nuclear transfection or cytoplasmic transfection. The expression of heparanase was detected by RT-PCR and Western blotting 48 h, 72 h and 96 h post-transfection. In addition, wound healing and invasion assays were performed to estimate the effect of TGS of the heparanase gene on the migration and invasion of hepatoma SMCC-7721 cells.

**Results:**

Protein and mRNA expression of the heparanase gene were interfered with by TGS or post-transcriptional gene silencing (PTGS) 48 h after transfection. At 72 h post-transfection, the expression of the PTGS group of genes had recovered unlike the TGS group. At 96 h post-transfection, the expression of the heparanase gene had recovered in both the TGS group and PTGS group. Invasion and wound healing assays showed that both TGS and PTGS of the heparanase gene could inhibit invasion and migration of hepatoma SMCC-7721 cells, especially the TGS group.

**Conclusions:**

TGS can effectively interfere with the heparanase gene to reduce the invasion and migration of hepatoma SMCC-7721 cells.

## Background

The heparanase gene is located on human chromosome 4q 21.3, which contains a CpG island at the 5′ end of the promoter region, indicating that expression of heparanase is regulated by epigenetic mechanisms such as methylation [1]. High levels of heparanase have been reported for hepatic carcinoma, breast cancer, colorectal cancer, lung cancer and lymphoma [2-4], and are involved in tumor metastasis and invasion [5-7]. On the other hand, inhibition of expression of heparanase has been reported to have an inhibitory effect on cancer invasion and metastasis. Zheng *et al*. reported that small RNA interference-mediated gene silencing of heparanase could inhibit the invasion, metastasis and angiogenesis of gastric cancer cells [8]. RNA interference (RNAi), a new technology in molecular biology, has become one of the most commonly used methods in research into gene function [9], including transcriptional gene silencing (TGS) and post-transcriptional gene silencing (PTGS) [10]. In PTGS, a small molecule RNA (siRNA) is designed for the coding region of a gene and targets the corresponding mRNA sequence-specific binding and degradation of the target sequence, inducing gene silencing [9]. Continuous activation of upstream gene transcription leads to synthesis of a large amount of mRNA, which makes it difficult to maintain the silencing of a gene for a long time. Therefore, the interference efficiency of PTGS is not high. Previous studies have found that TGS has a high efficiency in plant cells, acting through permanent gene silencing by DNA methylation or histone deacetylation of the 5′ end of the promoter region of DNA. This is called siRNA-direct DNA methylation or histone modification [11-13]. Kawasaki *et al*. [14] have found that this effect of TGS also occurs in human cells. Theoretically, interfering with a gene using an siRNA targeting the 5′ end of the promoter region can produce a long-lasting silence. Therefore, TGS seems more effective than PTGS with respect to the costs and prospects for clinical application.

In the present study, siRNA was transfected into hepatoma SMCC-7721 cells using TGS and PTGS to interfere with the expression of heparanase. Differences in the effect and the length of gene silencing were determined to evaluate the impact of silenced heparanase on the migration and invasion of SMCC-7721 cells.

## Methods

### Cell culture

Hepatoma SMCC-7721 cells (a hepatoma cell line) were purchased from the Institute of Biochemistry and Cell Biology of the Chinese Academy of Science in Shanghai. The cells were cultured in DMEM (Gibco, Carlsbad, CA, USA) supplemented with 10% fetal bovine serum (FBS) (Gibco, Carlsbad, CA, USA) at 37°C, 95% humidity and 5% CO_2_. The cells were passaged every 3 to 4 days by trypsinization.

### Preparation of siRNA and transfection

The complete genome of heparanase (GenBank accession: [GenBank:NC_010725]) was used to design the siRNA sequences. Both TGS and PTGS siRNA oligos were designed and synthesized by Bioo Scientific Corporation (Austin, TX, USA). The sequences were blasted with the promoter region or mRNA of the genome by DNAMAN software. The siRNA sequences of the oligos used in the following experiment were as follows: for TGS of heparanase: GAGGAAGUGCUAGAGACUCU and for PTGS of heparanase: CCUUAAGAAGGCUGAUAUU. Nucleofections were carried out as previously described by nucleofection with the Nucleofector device from Amaxa Biosystems (Cologne, Germany) [15]. The DharmaFECT transfection reagent (Thermo Scientific, Pittsburgh. PA, USA) was used for transfection of PTGS according to the manufacturer’s instructions.

### RT-PCR

At 48 h, 72 h and 96 h post-transfection, cells were harvested and total RNA was extracted using the TRIzol reagent (Life Technologies, Carlsbad, CA, USA) according to the manufacturer’s instructions. Complementary DNA (cDNA) was obtained by using a reverse transcription kit (Fermentas, Pittsburgh. PA, USA). The following primers were designed for RT-PCR: heparanase: sense: 5′-ATGTGGAGGAGAAGTTACGG-3′; antisense: 5′-TGAGTTGGACAGATTTGGAA-3′ and β-actin: sense: 5′-CATCCAGCGTACTCCAAAGA-3′; antisense: 5′-GACAAGTCTGAATGCTCCAC-3′. Amplifications were performed with 32 cycles of 30 sec at 94°C, 30 sec at 55°C and 45 sec at 72°C. The PCR product was then assessed by 1.2% agarose gel electrophoresis and visualized using the Gel Documentation System (BIO-RAD, Hercules, CA, USA). For real-time quantitative RT-PCR, the following primers for heparanase were designed: sense: 5′-GTGGTGATGAGGCAAGTATTC-3′ and antisense: 5′-GTGGTGATGAGGCAAGTATTC-3′. The mRNA of heparanase was determined with the EverGreen PCR kit (TransGen Biotech Co, Ltd, Beijing, China) using real-time RT-PCR (ABI7500, Carlsbad, CA, USA). The PCR mixture (20 μl final volume per reaction) was prepared according to the manufacturer′s protocol. PCR amplifications were performed with 40 cycles of 30 sec of denaturation at 94°C, 45 sec of annealing at 60°C and 45 sec of elongation at 72°C. Data were analyzed with the standard 2^-△△Ct^ method and values are expressed as the average of triplicates.

### Western blot

SiRNA-transfected hepatoma SMCC-7721 cells were harvested at 48 h, 72 h and 96 h post-transfection. The harvested cells were washed and lysed with lysis buffer (0.5% SDS, 1 mM tris–HCl, 1 mM sodium ortho-vanadate and protease inhibitor cocktail (Sigma, St. Louis, MO, USA)). The total protein concentrations were determined with the Bradford method [16]. An equal amount of protein from each subject was separated through SDS-PAGE and subsequently transferred onto PVDF (Polyvinylidene fluoride) membranes. The membranes were blocked with 5% (w/v) nonfat dry milk in tris-buffered saline with 0.05% Tween 20 for 1 h and followed with 1 h of incubation with mouse anti-sera against β-actin (1:2,000) or rabbit anti heparanase (1:500). HRP (horse radish peroxidise) -conjugated goat anti-mouse or rabbit immunoglobulin G (IgG) (1:1,000) was used as secondary antibodies and the reactions were detected using a chemiluminescence kit (Pierce, Rockford, IL, USA).

### Migration assay

To investigate the role of heparanase in the migration of hepatoma SMCC-7721 cells, a wound-healing test was performed. Seven groups of cells were used in this experiment: the control cells, TGS transfected cells (48 h, 72 h and 96 h post-transfection) and PTGS transfected cells (48 h, 72 h and 96 h post-transfection). Transfection for TGS and PTGS were carried out according to the process described above. The cells were separately seeded in 35 mm plates and cultured in DMEM with 10% FBS. A sterile 200 μl pipette tip was used to scratch the cell monolayers, creating a wound when the cells reached confluence. Images of the wounds were captured at the beginning of the assay and three times (48 h, 72 h and 96 h post-transfection) after the scratch during wound healing. Then the images were captured to compare the migration ability of the cells of each group. All samples for each group were assayed in duplicate. The experiment was performed three times.

### Invasion assay

To assess the role of heparanase in the invasion of hepatoma SMCC-7721 cells, an invasion assay was performed, using the BD BioCoat Matrigel™ invasion chambers (BD Biosciences, San Jose, CA, USA) pre-coated with BD Matrigel matrix (simulating the extracellular matrix) according to the manufacturer’s protocol. The medium of the transfected (TGS and PTGS) or untransfected cells was replaced with serum-free DMEM medium and cultured for 24 h at 37°C, followed by digestion with 0.25% trypsin and cell counting. We separately added 5 × 10^4^ control hepatoma SMCC-7721 cells, TGS transfected hepatoma SMCC-7721 cells and PTGS transfected hepatoma SMCC-7721 cells suspended in 100 μl serum-free DMEM medium onto the apical chambers carefully, and added 100 μl DMEM medium with 10% FBS to the basal chambers. If provided with sufficient nutrition, cells in apical chambers may migrate into the basal chambers. After 24 h of culture at 37°C, the chambers were washed twice and fixed with 1 ml of formaldehyde for 20 min. Then the chambers were stained with Gentian violet for 15 min followed by three washes. The cells were observed and counted using an inverted microscope (DMI4000B, Leica, Solms, Germany).

### Statistical analysis

Statistical analysis was performed using SPSS 13.0. The *in vitro* invasion assay was analyzed using a one-way ANOVA test. A *P* value (two-sided) of 0.05 was considered statistically significant.

## Results

### Transcriptional gene silencing and post-transcriptional gene silencing of heparanase mRNA in hepatoma SMCC-7721 cells

**Figure 1:**
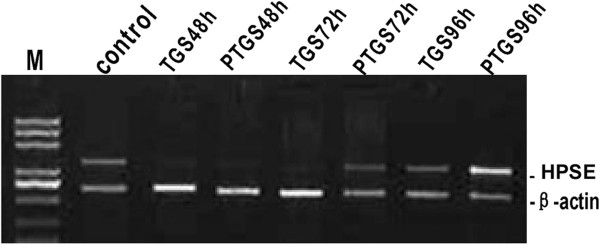
**Gene expression of heparanase in TGS and PTGS groups.** control: untransfected cells; HPSE, heparanase; M: DNA ladder; PTGS, post-transcriptional gene silencing; PTGS48h: 48 h after PTGS transfection; PTGS72h: 72 h after PTGS transfection; PTGS96h: 96 h after TGS transfection; TGS, transcriptional gene silencing; TGS48h: 48 h after TGS transfection; TGS72h: 72 h after TGS transfection; TGS96h: 96 h after TGS transfection.

Expression of heparanase mRNA was assessed by RT-PCR. As shown in Figure 1, the mRNA of the heparanase gene of the TGS group showed there was successful interference at 48 h and 72 h post-transfection. Unlike the TGS group, the heparanase expression of the PTGS group had recovered at 72 h post-transfection. The heparanase expression of both TGS and PTGS transfected hepatoma SMCC-7721 cells had recovered at 96 h post-transfection. Quantitative RT-PCR gave similar results for the expression of the heparanase gene: the heparanase gene was expressed in both the TGS and the PTGS groups and the level was nearly half that of the control group. These results indicate that both TGS and PTGS of heparanase can interfere with the expression of heparanase mRNA. However, silencing of heparanase only lasted for no more than 72 h using PTGS, indicating a weaker silencing effect compared with TGS.

### Transcriptional gene silencing and post-transcriptional gene silencing of heparanase protein in hepatoma SMCC-7721 cells

**Figure 2:**
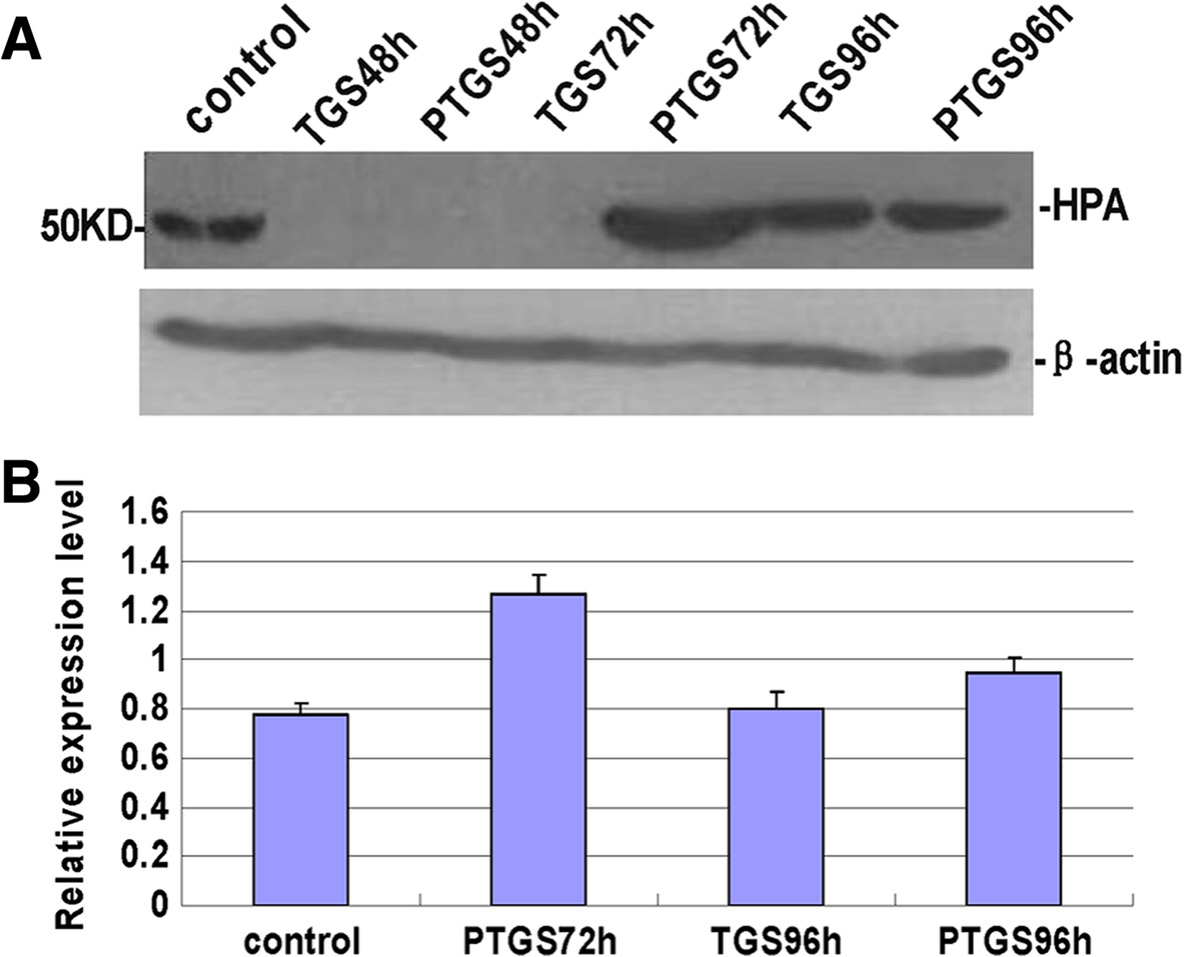
**Protein expression of heparanase in TGS and PTGS groups. (A)** Protein expression of heparanase in the TGS and PTGS groups. **(B)** Semi-quantitation of heparanase protein in the TGS and PTGS groups. control: untransfected cells; PTGS, post-transcriptional gene silencing; PTGS48h: 48 h after PTGS transfection; PTGS72h: 72 h after PTGS transfection; PTGS96h: 96 h after TGS transfection; TGS, transcriptional gene silencing; TGS48h: 48 h after TGS transfection; TGS72h: 72 h after TGS transfection; TGS96h: 96 h after TGS transfection. HPA, heparanase.

The expression of the heparanase protein was assessed using Western blotting and the results are shown in Figure 2. The heparanase expression of the TGS group was successfully interfered with at 48 h and 72 h post-transfection. The heparanase expression of the PTGS group had recovered at 72 h post-transfection. As with gene expression, the heparanase protein levels of both TGS and PTGS transfected hepatoma SMCC-7721 cells had recovered at 96 h post-transfection. These findings are consistent with those for mRNA expression.

### Migration of hepatoma SMCC-7721 cells after transcriptional gene silencing and post-transcriptional gene silencing of heparanase

**Figure 3:**
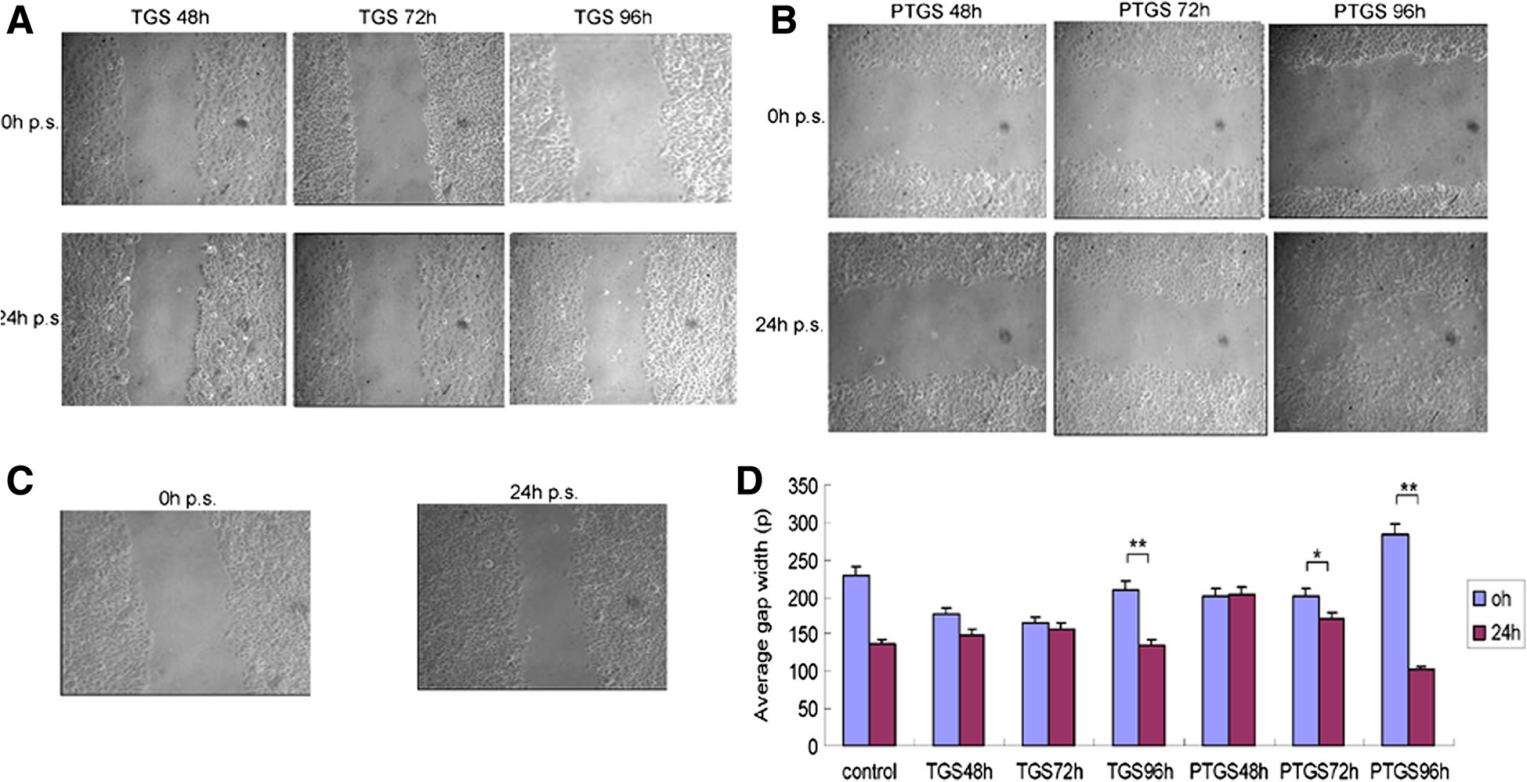
**Effect of TGS and PTGS of heparanase on migration of SMCC-7721 cells.** At 48 h, 72 h and 96 h after TGS or PTGS transfection, SMCC-7721 cells were seeded in 35 mm plates and scratched. Pictures of the TGS group **(A)**, PTGS group **(B)** and the control group **(C)** were taken 0 h and 24 h after the scratch and the average width of the gaps was then measured **(D)**. *P < 0.05 and **P < 0.01 compared with 0 h. control: untransfected cells; p.s., post scratch; PTGS, post-transcriptional gene silencing; PTGS48h: 48 h after PTGS transfection; PTGS72h: 72 h after PTGS transfection; PTGS96h: 96 h after TGS transfection; TGS, transcriptional gene silencing; TGS48h: 48 h after TGS transfection; TGS72h: 72 h after TGS transfection; TGS96h: 96 h after TGS transfection. **(D)** Quantitation of the average width of the gaps.

Compared with the control group, the wound gaps of the TGS group and the PTGS group were more evident after 48 h and 72 h of migration. On the other hand, the gaps for both groups became smaller after 24 h of migration, as bigger and longer cells gathered toward the gap center (Figure 3). The migration capability of the two groups of cells was enhanced significantly at 96 h post-transfection. In combination, these results suggest that both TGS and PTGS of heparanase gene could inhibit the migration of hepatoma SMCC-7721 cells. Moreover, TGS of heparanase had a longer inhibitory effect on cell migration than PTGS of heparanase.

### Invasion of hepatoma SMCC-7721 cells after transcriptional gene silencing and post-transcriptional gene silencing of heparanase

**Figure 4:**
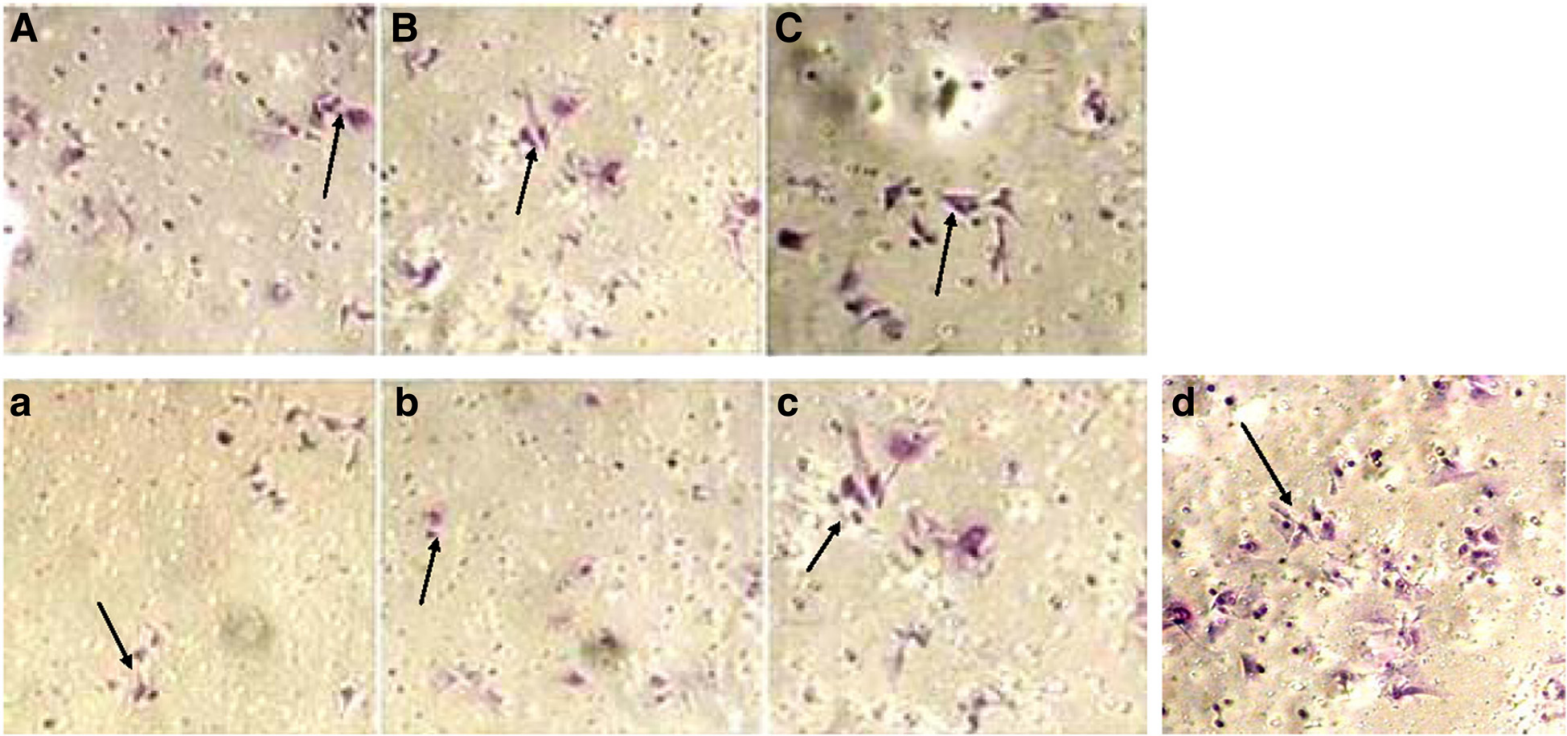
**Effect of TGS and PTGS of heparanase on invasion of SMCC-7721 cells. (A)** 48 h after TGS transfection. **(B)** 72 h after TGS transfection. **(C)** 96 h after TGS transfection. **(a)** 48 h after PTGS transfection. **(b)** 72 h after PTGS transfection. **(c)** 96 h after TGS transfection **(d)** Quantitation of the average width of the gaps. .

At 48 h after TGS and PTGS transfection, 50 and 78 cells, respectively, had passed through the fiber membrane at the bottom of the Transwell chamber. At 72 h after TGS and PTGS transfection, there were 60 and 88 cells, respectively. For the control group, 100 and 130 cells passed through the Transwell chamber fiber membrane, suggesting there was reduced invasion ability of the two transfected groups compared with the control group (Figure 4). At 96 h after transfection, 98 and 100 cells for the TGS and PTGS groups, respectively, had passed through the membrane. The results were not significant compared with the control group (count was 120, *P* > 0.05). Compared with the TGS group, the invasion ability of the PTGS group was much weaker at 48 h and 72 h after transfection (Table 1). However, the invasion ability of the TGS and PTGS groups showed no obvious differences at 96 h after transfection.

**Table 1:** Number of SMCC-7721 cells migrated across the membrane after TGS and PTGS of heparanase

**Time**	**PTGS (mean ± standard deviation)**	**TGS (mean ± standard deviation)**	**Control (mean ± standard deviation)**
48 h	3.33 ± 1.5*	1 ± 1*	14 ± 2.6
72 h	7.6 ± 1.5**	1.3 ± 0.68*	17 ± 1.5
96 h	12 ± 2.1	8.6 ± 1.5**	15 ± 1.3

*P < 0.05 and **P > 0.05 compared with the control group.

PTGS, post-transcriptional gene silencing; TGS, transcriptional gene silencing.

## Discussion

Invasion and metastasis are the main problems in the clinical treatment of tumors and heparanase is believed to play an important role in these processes. Currently, studies have paid more attention to gene therapy in the elucidation of the molecular mechanism of cancer. Recently, RNAi has been successfully used to interfere with the expression of a variety of target genes to inhibit the growth of different tumor cells. In addition to cancer genes, genes that express anti-apoptotic molecules, telomerase and growth factor receptors as well as some signaling molecules have also been interfered with in other anti-tumor studies [17,18]. RNAi has been used to explore the function and interaction of genes, providing a convenient way to screen the target genes of new drugs.

It has been proved that RNAi-induced heparanase silencing can inhibit cell invasion of gastric carcinoma [19], hepatocellular carcinoma [20] and embryonal rhabdomyosarcoma [21]. Traditional gene-targeting therapy for cancer is at the post-transcriptional level, silencing the corresponding mRNA. However, the promoter of the gene is still active, which creates difficulties in maintaining long-term silencing of the transcription of the target genes. To keep a target gene silent, the only approach is the continuous addition of siRNA. In human cancer gene therapy, there are still many problems, such as finding the right vector for gene therapy. Significant effort has been expended in establishing a stable and specific therapeutic gene vector for expression in tumor cells that can be used in clinical treatment. However, the technology is not mature yet. Drugs for clinical experimental treatments synthesized *in vitro* by chemical modification of an oligonucleotide are expensive, have a short half-life and are sometimes cytotoxic especially when administered over a long period of time. Transcriptional-level RNAi-induced gene silencing not only regulates the degradation of homologous sequences but also induces heterochromatin formation, DNA methylation and histone modification, so that the gene cannot be transcribed. In theory, once an siRNA for the 5′ end promoter has been administered, the gene can be permanently silenced. Therefore, there is a broad application potential for RNAi at the level of transcription. This study was a comparative study to compare the effect of gene interference of TGS with that of PTGS to confirm the superiority of TGS interference.

First, siRNAs that targeted the promoter region within the nucleus were designed. Corresponding siRNAs targeting the cytosolic mRNA region were also designed. PCR analysis showed that genes for both of the two groups of cells were silenced 48 h after siRNA transfection. However, at 72 h after siRNA transfection, the interference seen in the PTGS group had significantly decreased, while the interference seen in the TGS group was still obvious. At 96 h after transfection, the interference seen in the TGS group had declined as well. The protein expression of heparanase was similar. Western blot experiments showed that the silence time of heparanase in cells from the TGS group was much longer than that of the PTGS group. However, both the TGS and PTGS groups showed slight inference at 96 h after transfection. Wound healing and Transwell invasion assays also proved that there was less invasion and migration of cells for both the TGS and PTGS groups within 48 h of transfection. At 72 h after transfection, the migration and invasion capacity of the PTGS group had recovered a little, while the TGS group showed no obvious difference from the situation 24 h before. At 96 h after transfection, the migration and invasion capability of cells in the PTGS group had been restored significantly. Though the TGS group expressed heparanase, the level was still very low. In combination with the experimental results above, a much longer silence time of heparanase was observed in the TGS group with respect to the PTGS group. However, TGS did not achieve a permanent gene knockout as previously expected.

Although this study could not confirm a permanent knockout of the heparanase gene for either the gene level or protein levels, the silence time of heparanase for the TGS group was longer than that of the PTGS group. Wound healing and Transwell invasion assays also proved that migration and invasion of the TGS cells were reduced. There was still a wide gap between our results and the speculation that TGS may knock out gene expression permanently. There are three possible reasons. The first is the low efficiency of nuclear transfection. Only a few of the siRNAs were transfected into the nuclei by electric transfection and the majority of the siRNAs degraded in the cytoplasm. This may have caused the unexpected results seen in our study. In preliminary experiments, ordinary liposome transfection was used and there was almost no interference after TGS while PTGS produced significant genetic interference (data not shown), indicating that effective nuclear transfection is important for TGS. Second, different effects may have occurred because of the different type of cells or genes used for TGS. Epigenetics may affect the efficiency of nuclear transfection. Third, gene expression controlled by epigenetics is reversible.

## Conclusions

We found that TGS could effectively interfere with the heparanase gene to reduce the invasion and migration of hepatoma SMCC-7721 cells, which is a very important experimental basis for TGS research in cancer gene therapy. The mechanism of TGS, as well as whether it is more effective for the RNAi to target CpG islands or transcription factor binding sites are still unknown. How long can RNAi-induced epigenetic alterations be maintained? Whether CpG islands in genes are suitable as the promoter of RNAi is still unclear. Therefore, further research is needed for these problems.

## Abbreviations

DMEM: Dulbecco’s modified Eagle’s medium; FBS: fetal bovine serum; IgG: immunoglobulin G; PTGS: post-transcriptional gene silencing; RNAi: RNA interference; RT-PCR: reverse transcriptase polymerase chain reaction; siRNA: small interfering RNA; TGS: transcriptional gene silencing.

## Competing interests

The authors declare that they have no competing interests.

## Authors’ contributions

WX and CZ designed the study and drafted the manuscript. PG, HZ, WD, SS, QL and JZ did the experiments. WX, GL, SZ and MY collected and analyzed the data. All authors read and approved the final manuscript.
